# Student perceptions of gamified audience response system interactions in large group lectures and via lecture capture technology

**DOI:** 10.1186/s12909-015-0373-7

**Published:** 2015-05-22

**Authors:** Robin K Pettit, Lise McCoy, Marjorie Kinney, Frederic N Schwartz

**Affiliations:** A. T. Still University, School of Osteopathic Medicine in Arizona, 5850 E. Still Circle, Mesa, AZ 85206 USA

**Keywords:** Audience response system, Clicker, TurningPoint, Medical microbiology, Game, Gamification

## Abstract

**Background:**

Higher education students have positive attitudes about the use of audience response systems (ARS), but even technology-enhanced lessons can become tiresome if the pedagogical approach is exactly the same with each implementation. Gamification is the notion that gaming mechanics can be applied to routine activities. In this study, TurningPoint (TP) ARS interactions were gamified and implemented in 22 large group medical microbiology lectures throughout an integrated year 1 osteopathic medical school curriculum.

**Methods:**

A 32-item questionnaire was used to measure students’ perceptions of the gamified TP interactions at the end of their first year. The survey instrument generated both Likert scale and open-ended response data that addressed game design and variety, engagement and learning features, use of TP questions after class, and any value of lecture capture technology for reviewing these interactive presentations. The Chi Square Test was used to analyze grouped responses to Likert scale questions. Responses to open-ended prompts were categorized using open-coding.

**Results:**

Ninety-one students out of 106 (86 %) responded to the survey. A significant majority of the respondents agreed or strongly agreed that the games were engaging, and an effective learning tool. The questionnaire investigated the degree to which specific features of these interactions were engaging (nine items) and promoted learning (seven items). The most highly ranked engagement aspects were peer competition and focus on the activity (tied for highest ranking), and the most highly ranked learning aspect was applying theoretical knowledge to clinical scenarios. Another notable item was the variety of interactions, which ranked in the top three in both the engagement and learning categories. Open-ended comments shed light on how students use TP questions for exam preparation, and revealed engaging and non-engaging attributes of these interactive sessions for students who review them via lecture capture.

**Conclusions:**

Students clearly valued the engagement and learning aspects of gamified TP interactions. The overwhelming majority of students surveyed in this study were engaged by the variety of TP games, and gained an interest in microbiology. The methods described in this study may be useful for other educators wishing to expand the utility of ARS in their classrooms.

**Electronic supplementary material:**

The online version of this article (doi:10.1186/s12909-015-0373-7) contains supplementary material, which is available to authorized users.

## Background

According to Prince [1], the core elements of active learning are the introduction of activities into the traditional lecture, and the promotion of student engagement. Audience response systems (ARS), or clickers, are active learning tools that involve most to all students in a classroom. ARS were designed to electronically poll large groups, allowing individual responses on hand-held keypads, and immediate reporting of aggregated results (feedback) in graphic form. Although ARS have been available for decades, widespread use in higher education wasn’t until 2003 [2]. As summarized in several useful review articles [2–6], higher education students have positive attitudes about the use of ARS, and perceive that they are more attentive and engaged when ARS is used during lectures.

It is widely recognized that active learning yields positive learning outcomes [1, 7–9]. Students who engage interactively with each other and with the instructor learn concepts better, retain them longer, and can apply them more effectively in other contexts than students who sit passively listening [7, 8, 10]. Most health professions education students perceive learning benefits with ARS, and meta-analysis suggests that ARS’ impact on learning outcomes is neutral to moderately beneficial [6]. The magnitude of the ARS effect depends on a number of factors, particularly the intervention against which ARS is compared. Not surprisingly, studies comparing ARS to other interactive teaching modalities showed less impact on knowledge outcomes than those comparing ARS to non-interactive teaching [6]. Future research using randomized methods should help elucidate the learning outcome benefits of ARS.

ARS are considered useful instructional tools because they increase interaction between faculty and students, allow for formative assessment of student knowledge, maintain students’ attention during lectures, and focus students’ attention on key points [11]. Multiple studies have shown that students feel engaged during ARS interactions [12–16]. However, as indicated by Patterson et al. [17] and Kay and LeSage [2], the specific features of these interactions that are engaging for students are not well described.

The core elements of active learning, student activity and engagement, are central to educational game theory [18–21]. Games are growing in popularity at all levels of education, including medical education [22–35], and include simulations, virtual environments, social and cooperative play, and alternative reality games [18]. A survey of family medicine and internal medicine residency programs directors in the United States indicated that 80 % used games as an educational strategy in their residency training programs [23]. While there is evidence that students find games more enjoyable and stimulating than standard lectures [33, 35], evidence for their utility in increasing knowledge is conflicting, perhaps in part due to the limited number of rigorous studies [18].

Gamification is the integration of gaming elements, mechanics and frameworks into non-game situations and scenarios [36]. The purpose of gamification is to make routine activities fun, interesting and addictive [20, 37]. When designing learning games, educators can draw from the corporate world’s ‘playbook’ of game dynamics by incorporating game pleasure elements such as challenge, surprise, and pride in accomplishment, and game mechanics such as rules, competition and immediate feedback [20, 37].

We introduced multiple game elements and mechanics [20, 37] into year 1 medical microbiology TurningPoint (TP) ARS presentations (detailed under **Methods**), and explored explanations for student engagement. Literature regarding gamification of TP is scarce. Very recently, Schlegel and Selfridge [38] reported the use of audience response technology to implement a team review game on the last day of a Dermatology course. The authors state that the game promoted learning and student satisfaction, but no data are provided. To our knowledge, the engagement aspects of gamified TP interactions have not been studied.

As Cain and Robinson [4] discussed, clickers do not magically transform a classroom; how they’re used determines their effectiveness. Therefore, the aim of this study was to provide insight into best practices for ARS engagement in a year 1 osteopathic medical student (OMS1) large group classroom setting. This study comprises part of an A.T. Still University School of Osteopathic Medicine in Arizona (ATSU SOMA) goal to improve technology-enhanced active learning in our curriculum [39]. With the goal of increasing engagement in large group medical microbiology sessions, we developed and implemented a variety of TP interactions, including game interactions, during academic year 2013/2014 (Class of 2017, year 1). Our hypothesis was that students would be engaged by the variety of TP interactions. The following research questions were posed: In which ways do TP games foster engagement? Do students perceive any learning benefit from the TP games? Are there gender differences between student perceptions of the TP games? How do students use the questions from TP games after class? Do students who review the TP games via lecture capture (video/audio recording of large group lectures that students can view on their personal devices) perceive learning gains or assign any engagement value?

The results of this investigation begin to address areas of TP ARS research that have not been well-studied: gamification, specific engagement features of gamified TP ARS interactions, use of TP questions after class, and use by students that review using lecture capture.

## Methods

### Audience response systems

Wireless keypads (clickers) and a receiver were purchased from TurningPoint Technologies (Youngstown, OH). Clickers (ResponseCard® NXT) for each student in the class of 2017 were purchased by ATSU SOMA. All TP interactions for the present study were implemented in the anonymous mode. Student clicker numbers, not student names, were displayed on competition slides. There were no points/grades associated with the TP ARS activities.

### Construction of the TP games

TurningPoint operates as a PowerPoint plug-in. A menu for customizing TP interactions appears when TurningPoint is selected from the menu in PowerPoint. Pull-down tabs can be used to create different types of questions (true/false, multiple choice, short answer, numeric response), insert objects (custom correct answer indicator, various types of summary charts, countdown timer), build in competition (team assignment, team leader board, team Most Valuable Player [MVP], participant leader board, fastest responder, wager), and create peer teaching slides (comparative links). Images, video and audio can be imported into TP slides. The questions in this study ranged from basic knowledge to clinical vignettes requiring application and analysis. The majority of the questions were clinical vignettes with multiple choice answers, mimicking the style of the United States Medical Licensing Examination (USMLE) and the Comprehensive Osteopathic Medical Licensing Examination of the United States (COMLEX-USA). For individual and team competitions, individual participant boards or team leader boards were spaced at intervals throughout a presentation to track individual clicker ID numbers and scores, or team names and scores.

**Fig. 1:**
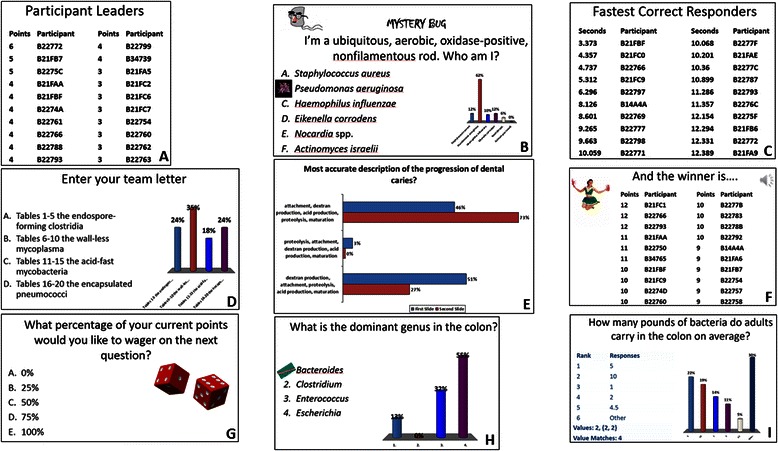
TP slides showing examples of gamification elements and the variety of TP ARS interactions. (**a)** participant leaderboard (**b**) mystery bug question (image used with permission from S.E. Gould, http://blogs.scientificamerican.com/network-central/2011/10/11/introducing-sciamblogs-bloggers-s-e-gould/); correct answer indicator (microbe image from CDC Newsroom Image Library) (**c**) fastest correct responder (**d**) team competition (**e**) peer teaching (**f**) winner of individual participant competition (cheerleader image from FreeDigitalPhotos.net) (**g**) wagering (dice image by Stephen Silver at Open Clip Art Library via Wikimedia Commons) (**h**) identifying misconceptions prior to introducing new material; correct answer indicator (microbe image by Janice Haney Carr at Public Health Image Library) (**i**) ranking responses

Games can be defined as outcome-oriented activities that proceed according to a set of rules and often involve decision making [40]. Outcome or goal-oriented activities are salient to adult learners [41], and specifically, to medical students (McCoy et al., submitted). TP ARS interactions inherently have a few game elements and mechanics, for example, the action of clicking the device and the real-time information flow (immediate feedback). We introduced many more game elements and mechanics [20, 37] into our TP ARS large group interactions: *rules, objects* (e.g. leader boards inserted at intervals to show points accumulated during the game by each player or team (Fig. 1a), custom correct answer indicators representing the organism being discussed (Fig. 1b), mystery bug character for rapid review (Fig. 1b)); *action* using fastest responder slides inserted after simple recall-type questions (Fig. 1c); *collaboration* using team competitions where students join teams at the game outset by clicking on their team letter (Fig. 1d); *peer teaching* where students answer a question individually using their clickers, then discuss the question with a neighbor, and answer the question again using any knowledge gained (Fig. 1e); *individual and team competition* (individual or team leader boards (Fig. 1f shows an individual leader board at the end of a gamified TP ARS interaction)); *social fabric* (the idea that people like one another better after they’ve played games together, build a higher level of trust, and have a greater willingness to work together); *progression dynamic* (individual or team participant boards inserted at intervals); *countdown* (fastest responder); *urgent optimism* (desire to act immediately to tackle an obstacle combined with the belief that we have a reasonable hope of success); *achievement, status* (winner of individual participant board or fastest responder or team MVP); *prizes* (trivial prizes, for e.g., candy in the shape of the microbe(s) covered in that interactive lecture); and *chance* (the risk of wagering; these slides were occasionally inserted prior to the final question (Fig. 1g)). Game pleasure elements [20] included *sensation* (imported audio of clapping when winning score displayed on screen (Fig. 1f, upper right), imported images, holding the clicker), *challenge* (most questions were in the style of United States medical licensing exams), *anticipation* (students told at beginning of lecture that there would be a certain number of fastest responder slides, or that in team competition there would be a MVP that day), *humor* (images, team names), *surprise* (random insertion of fastest responder slides, wager slides and tie-breaker slides), and *pride in accomplishment* (occasional simple questions to build confidence).

Each medical microbiology presentation incorporated multiple game elements and mechanics, but care was taken to vary the game elements and mechanics from presentation to presentation. To provide a few examples, within a single organ system course, only one medical microbiology presentation would incorporate a team competition, and custom correct answer indicators were different from presentation to presentation because they represented the microbe(s) being discussed.

### Development of the survey instrument

An original, 32-item questionnaire was used to measure student perceptions of the design features, engagement value, and learning value of the TP games (Additional file 1). To develop the survey, authors reviewed the TP literature, and developed items to investigate domains related to game design, engagement and learning.

#### Game design.

The domain “game design” investigated the TP game elements and mechanics described above under *Construction of the TP games*.

#### Engagement.

The domain “engagement” included sub-constructs such as learner-centered emotions [42], flow (energy level, enjoyment, focus) [43–45], and fun related to game activities such as holding a game device, the feeling of being in game mode, staying on one’s toes, or anticipating a prize.

#### Learning.

The domain “learning” focused on the value of games for critical thinking [42], connections among concepts, prioritization of concepts to review, and practice applying theoretical concepts. Other sub-concepts included challenge, peer-learning, and learning variety [19–21].

Three questions at the beginning of the survey addressed age range, gender, and whether the student typically viewed the TP presentations in person or via lecture capture. Students were asked to evaluate the extent to which they agreed with the statements using a Likert 5-point rating scale (1, Strongly Agree; 2, Agree; 3, Neutral; 4, Disagree; 5, Strongly Disagree or 1, Very; 2, Somewhat; 3, Neutral; 4, Not very; 5, Not at all). Likert scale questions and two open-ended questions addressed the research questions. One of the open-ended questions solicited information on strategies for studying following TP games, and the other probed any learning value of the TP games when reviewed via Echo360 lecture capture. The ATSU Institutional Review Board (IRB) deemed the study exempt from IRB reporting requirements for human subjects research.

### Participants and setting

This study was conducted among a stable sample of 106 first-year medical students at ATSU SOMA during the 2013–2014 academic year (Class of 2017). Twenty-two gamified TP ARS microbiology presentations were offered during this period; six sessions in the summer of 2013, eight sessions in the fall of 2013, and eight sessions in the spring of 2014. Students were surveyed after completion of these sessions, several days prior to the final exam in their last organ system course.

### Echo360 lecture capture

Attendance for large group sessions, where the TP interactions were offered, is optional because Echo360 lecture capture technology is used in our medical education program.

### Data collection

Survey data collection involved an email solicitation containing a clickable link to an online survey. Students received the email survey during an unrelated large group session given by a faculty member who was not involved in the TP games. The students were given approximately 10 min to complete the survey on their personal devices during this class. Participation was voluntary and anonymous. Students were not asked to provide evidence of completion, and there were no rewards offered for completing the survey.

### Data analysis

Statistical analyses were completed using the statistical analysis software IBM SPSS Statistics 21^TM^. All Likert survey responses were categorized into either positive or neutral/negative responses, combining “strongly agree” and “agree” into the positive category, and “neutral”, “disagree” and “strongly disagree” into the neutral/negative response. These category responses were analyzed to determine if the proportion of positive and negative responses were the same across gender and lecture capture usage (Fisher’s Exact Test for outcomes where more than 20 % of the responses had counts less than 5 and Chi Square Test for all others).

We used open-coding [46] to analyze student responses to open-ended survey prompts. One author reviewed all comments and placed them in categories, and a second author reviewed and verified the categories.

## Results

A total of 91 students (86 %) in the Class of 2017 completed the TP game survey. There were 45 male respondents and 46 female respondents. There were 58 respondents age 22–25, 27 age 26–30, and 6 age 31–35. The survey instrument generated both Likert scale and open-ended response data to address the research questions. Students’ perceptions of the TP ARS sessions were queried in four areas: game design and variety, engagement, learning, and any value of the TP interactions via lecture capture.

### Game design and variety

**Fig. 2:**
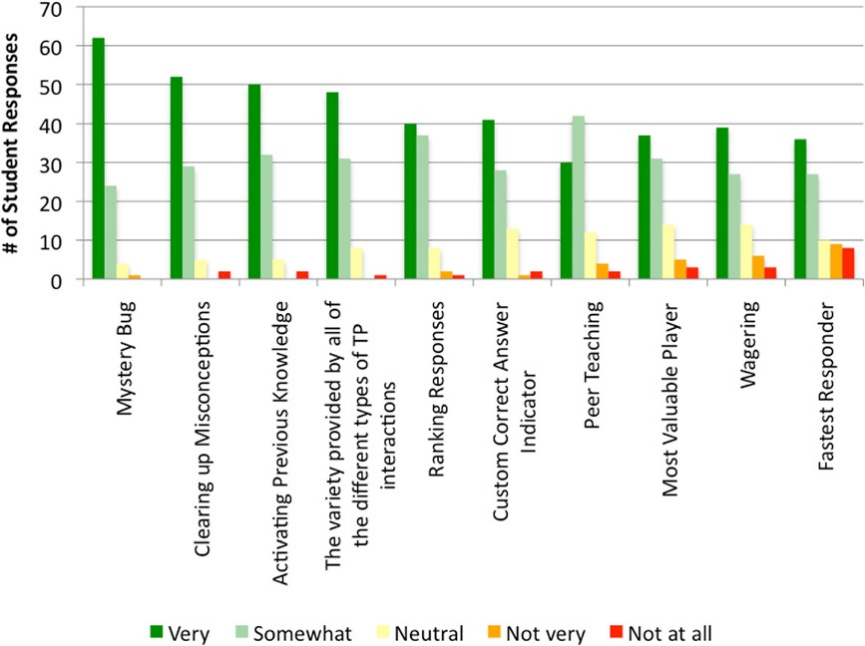
Summary of student responses to the prompt *Look at the photographs of the different types of TP interactions. Please rate the extent to which each activity is engaging*

During the survey, students were provided a single page with images of the various TP interactions. These were either examples of game elements and mechanics (Mystery Bug (Fig. 1b), Custom Correct Answer Indicator (Fig. 1b, 1h), MVP, Wagering (Fig. 1g), and Fastest Responder (Fig. 1c)), or they probed the variety incorporated into our TP interactions (Clearing up Misconceptions (Fig. 1h), Activating Previous Knowledge, Ranking Responses (Fig. 1i), Peer Teaching (Fig. 1e) and the Variety Provided by all of the Different Types of TP Interactions). In response to the prompt, *Look at the photographs of the different types of TP interactions. Please rate the extent to which each activity is engaging,* respondents indicated that the most engaging TP interactions were Mystery Bug, Clearing up Misconceptions and Activating Previous Knowledge (Fig. 2). At least 80 % of the respondents indicated that the Variety, the Ranking Responses and the Custom Correct Answer Indicator were very or somewhat engaging, and 74-79 % of the respondents found the Peer Teaching, MVP and Wagering slides very or somewhat engaging. The item with the lowest engagement ranking was Fastest Responder; 70 % of respondents found these interactions very or somewhat engaging. Six students wrote comments in response to the prompt *Other Comments* in this section of the survey*: I love team games; TP is an awesome way to keep students engaged during lectures!; I love the custom correct answer indicator; TP allows me to stay connected to the lecture and solidifies the information that I learn; The mystery bug slides were the best!!! Helped clarify concepts and focus my studying; Love the TP activities! Thanks for putting in the time/effort to make these activities!*

### Engagement

**Fig. 3:**
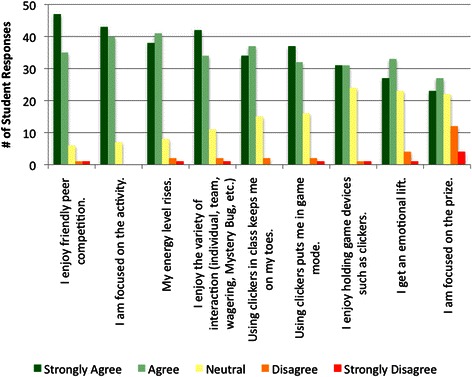
Summary of student responses to the prompt *Engagement/Flow: Please rate the extent to which you agree with the following statements. During TP games*…

One set of questions probed the engagement value of the various game elements and mechanics. In response to the prompt *Engagement/Flow: Please rate the extent to which you agree with the following statements. During TP games*…at least 85 % of the respondents agreed or strongly agreed that they enjoyed the friendly peer competition, were focused on the activity, their energy level rose, and they enjoyed the variety of interactions (this prompt included the game examples Individual, Team, Wagering, Mystery Bug) (Fig. 3). In response to specific prompts about the clickers, 79 % of the respondents agreed or strongly agreed that using clickers in class kept them on their toes, 77 % agreed or strongly agreed that using clickers put them in game mode, and 69 % agreed or strongly agreed that they enjoyed holding game devices such as clickers. Sixty-eight percent of the respondents agreed or strongly agreed that during TP games they got an emotional lift. Only 55 % of the respondents agreed or strongly agreed that they were focused on the prize during TP games, the lowest positive response in the engagement category. Rewards were nominal, typically candy in the shape of the microbe(s) covered during that class session. However, for some team competitions, a book or flashcard set was raffled off amongst the winning team.

In response to the question, *In which of the following TP environments do you learn the best?*, 53 % of the students selected individual competition, one clicker per student; 33 % selected team competition, one clicker per student; 9 % selected team competition, one clicker per team; and 5 % selected no competition.

### Learning

**Fig. 4:**
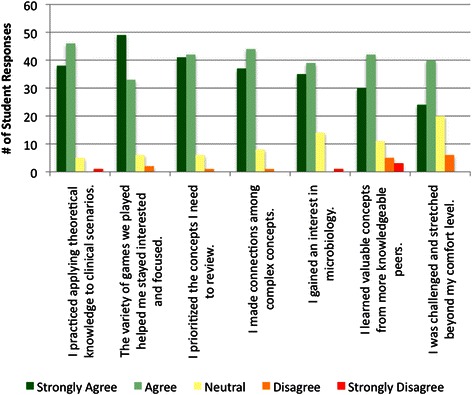
Summary of student responses to the prompt *Learning: To what extent did TP games foster Learning? During TP games*…

In response to the prompt *Learning: To what extent did TP games foster learning? During TP games*…at least 90 % of the respondents agreed or strongly agreed that they practiced applying theoretical knowledge to clinical scenarios, prioritized the concepts they needed to review, and made connections among complex concepts (Fig. 4). Ninety-one percent of the respondents agreed or strongly agreed that the variety of TP games played helped them stay interested and focused. Eighty-two percent of the respondents agreed or strongly agreed that they gained an interest in microbiology. Eighty percent of the respondents agreed or strongly agreed that they learned valuable concepts from more knowledgeable peers during the games. Opportunities for learning from peers included looking at the graphic results slide displayed after each question, team competitions with a single clicker, and peer teaching comparative link slides. Seventy-one percent of respondents agreed or strongly agreed they were challenged and stretched beyond their comfort level, the lowest positive response in the learning category. One learning subscale item on the survey showed a difference in responses between males and females. *I prioritized the concepts I needed to review* was ranked more positively by females (97.8 %) than by males (84.4 %), *p* = 0.030.

There were 49 responses to the open-ended question, *After TurningPoint games, my strategy for follow-up up studying involves…* These specific survey comments were categorized by theme using open coding [46] (Table 1). Key themes were Focus on weak areas, Review the game, Review the lecture and Make a study guide or chart. Ten students commented that they used the TP games to focus on weak areas, for example, one student used the TP questions for *Reviewing any gaps in knowledge elucidated by the questions.* Eighteen students commented that they reviewed the TP games while studying, for example, *Reviewing the same questions that were asked during class gives me a good idea of where I stand with the material. I can gauge where I stand with each lecture and it encourages my studying.* Since the TP questions were not provided before or after the in-class games, students had to access Echo360 in order to review the TP questions. Comments that coded with Review the lecture and Make a study guide or chart were not specific to TP. One comment under Miscellaneous revealed another way students might use TP questions for study: *I know how to prioritize my studying after seeing what the professor points out. It gives me a mental framework of how to study.*

**Table 1:** Responses to *After TurningPoint games, my strategy for follow-up studying involves*…, categorized by theme using open coding [46]

Theme	Specific Statement
Focus on weak areas	Study missed material.
	Reviewing what I didn't previously understand.
	Using the questions I missed to focus on weakness.
	Reviewing any gaps in knowledge elucidated by the questions.
	Returning to concepts in which I did not excel during clicker moments.
	Emphasizing the topics where I lacked knowledge the most.
	Focusing on weak areas.
	Reviewing the bugs and focusing on the ones I didn't understand.
	Reviewing bugs/questions I didn't know the answers.
	Going over concepts that were discussed during TP sessions that I realized I was not strong in.
Review the game	Reviewing the topics tested in the questions.
	Working on studying thru those questions.
	Going back over the TP questions on my own.
	Reviewing the same questions that were asked during class gives me a good idea of where I stand with the material. I can gauge where I stand with each lecture and it encourages my studying.
	Using the TP games to review and assess how much I retained and understood the material.
	Also gives me a guidance on what to study, but the highlighted information in lecture is more helpful. (*also fits under Review lecture*)
	Review the questions.
	Reviewing the questions before a test and trying to answer them on my own.
	Review TP questions to ensure understanding.
	Relook at them.
	Reviewing what was in the game first, it helps set priorities.
	Reviewing TP questions before an exam.
	Recalling the questions asked and reviewing those topics.
	Reviewing the lectures and the questions presented in TP. (*also fits under Review lecture*)
	Looking at answers for the TP questions.
	Reviewing the questions and highlighting important words.
	Reviewing all the material, but reviewing some material that was on the games. (*also fits under Review lecture*)
	Reviewing my notes with powerpoints constructed from the questions.
	I copy the screen of the question and answers, and look at them during studying time.
Review the lecture	Reviewing the powerpoints/key concepts.
	Reviewing the lecture slides, including the question slides. (*also fits under Review the game*)
	Review the lecture, study notes.
	Studying all of the material like I was going to in the first place.
	Going through slides.
	Reviewing material.
	Re-studying slides and writing out the bugs.
	Looking at the highlights from the bugs.
	Review.
	Reviewing bugs.
	Reviewing the lectures.
	Study in general since I'm never ready for the games.
Make a study guide or chart	Pay attention to key points and gather information for a study guide.
	Creating a study guide and reviewing it. I also quiz my classmates with hypothetical clinical scenarios.
	Reviewing the differences between the bugs and making charts.
	Making charts about all of the bugs.
Miscellaneous	Nothing. I do not look at the questions again.
	No change.
	I love these classes and try to always win the TP competitions!
	Pay attention in class and to details that differentiate the bugs.
	I know how to prioritize my studying after seeing what the professor points out. It gives me a mental framework of how to study.

### Value via lecture capture

The survey question, *I typically experienced TurningPoint presentations by this professor…* had two response options, *In person* or *Via lecture capture (Echo 360).* Sixty percent of the respondents indicated *In person*; 40 % of the respondents indicated *Via lecture capture*. Under *Other (please specify)*, one student submitted the response, *About half and half*. No Likert scale questions showed a significant difference in proportion of positive and negative responses between the two groups of students. Information gathered in the course of regular educational activities (course evaluations for eight organ system courses that ran from July, 2013 through May, 2014) for the Class of 2017 indicated that the percent of students who always relied on Echo360 instead of attending lectures ranged from 4 % in July, 2013, to 22 % in May, 2014. Since the survey was conducted in May, students who responded *Via lecture capture* in response to the prompt, *I typically experienced TurningPoint presentations by this professor*…, likely had more regular attendance in large group sessions, including interactive microbiology TP sessions, earlier in the year.

The second open-ended question probed the value of lecture capture (Echo360) for viewing gamified TP presentations. Forty-two students provided responses to the open-ended question, *Describe the learning value, if any, of viewing these TurningPoint presentations on Echo*. Responses were categorized by theme using open coding [46]. Key themes were Self-pace, Pause, Review/Repeat/Practice/Helpful, and Engagement (Table 2). Most of the responses were extremely positive, providing insight as to why students felt lecture capture was valuable for TP. Students valued the ability to do the TP questions at their own pace (Self-pace), pause the Echo for TP questions (Pause) and use the TP questions to focus on important information (Review/Repeat/Practice/Helpful, *Positive*). One student did not value the TP questions during the presentation because they hadn’t had enough time to study the material (Review/Repeat/Practice/Helpful, *Negative*). Five students wrote comments indicating that Echo had engagement value (Engagement, *Positive*), including the statement *I still find it useful, since I get to shout at my screen, “No, you silly people! The answer is C! C!” when people get the question wrong.* Eight students indicated that viewing the TP presentations on Echo was not as engaging as participating in class (Engagement, *Negative*). Some of the comments provided insight into why they didn’t find it as engaging, including, *it definitely is more valuable in person just based on the atmosphere it creates; it’s definitely not as engaging and fun; on Echo the competition drive was missing*. While some responses appeared to be written by students who solely used Echo360, and others appeared to be written by students who attended class and then viewed the presentation again using Echo360, we are not able to sort the responses. Nevertheless, the comments provide insight into how students use lecture capture for reviewing TP presentations, and their perceptions of any engagement value.

**Table 2:** Responses to *Describe the learning value, if any, of viewing these TurningPoint presentations on Echo,* categorized by theme using open coding [46]

Theme	Specific Statement
Self-pace	I enjoy the TP questions on Echo; I pause the presentation so that I can answer it myself before hearing the correct answer. I also like listening on echo because I can read the question at my own pace and not feel rushed to respond. (*also fits under Pause*)
	It is much much much faster.
	It's better on Echo because I can go at my own pace and skip through all of the wait time.
Pause	I can pause the question and think about it before answering the question. Sometimes, I would have screen shot these questions and reviewed them before the exam.
	I paused and thought of it myself.
	I would pause the presentation and answer the questions.
	Tons, allows me to pause and review concepts during the questions.
	I can pause the slide and review what was presented.
Review, Repeat, Practice, Helpful	I can assess my understanding of the lectures with the individual questions.
	When echoing, I use the TPs as practice questions.
	Absolutely loved the questions because they really made me evaluate if and how much information I really understood.
Positive	I still quiz myself during Echo, so I receive the same benefit.
	Helpful way to make sure I'm caught up with material.
	It's a great way to learn even on Echo where you can't participate.
	Good way to test my understanding of the lecture.
	Helps in knowing what to focus on.
	Huge learning value to integrate in and translate knowledge.
	Helps in pointing out important points.
	Repeating material.
	Even though not playing a game was still helpful.
	Still was very helpful, despite not being in class.
	It helps reinforce important information.
	It allows me to go through the same process without clicker in hand.
	Different way to learn is nice. Thank You.
	I enjoyed the questions they helped for instant review.
	I copy the screen of the question and answers, and look at them during studying time.
	Use it to see if I have retained and learned the material necessary to answer the question, and breaks up the monotony of just listening to the lecture.
Negative	Not good directly after presenting material because I haven't studied the material yet.
	They still helped me but not as much as going to class. (*also fits under Review, Repeat, Practice, Helpful, Positive*)
Engagement	I still find it useful, since I get to shout at my screen, "No, you silly people! The answer is C! C!" when people get the question wrong.
Positive	
	Definitely motivates and challenges/stimulates the brain to do well, know the material well, and study harder. (*also fits under Review, Repeat, Practice, Helpful, Positive*)
	Still very helpful because it's interactive and tests your knowledge.
	It helps review and understand the material better during class. Also, it keeps me engaged. (*also fits under Review, Repeat, Practice, Helpful, Positive*)
	Love these on Echo! I stop the Echo and answer as if I were in the classroom. Very useful and engaging!!!
Negative	While there isn't the same interaction, I still appreciate the evaluation of my current understanding of the topics. (*also fits under Review, Repeat, Practice, Helpful, Positive*)
	I get more out of it if I pause and try to answer for myself, but it definitely is more valuable in person just based on the atmosphere it creates. (*also fits under Pause*)
	On Echo, it's definitely not as engaging and fun. On Echo, I typically just run through it and copy answers. It doesn't challenge me as much, therefore, I don't learn as well...so with this professor, I tried my best to attend class.
	Not very useful for Echo, I try to play along.
	I would still try to answer the questions, but was not as engaged.
	Helps focus my studying, but other than that it has little value on Echo. (*also fits under Review, Repeat, Practice, Helpful, Positive*)
	Little value. I have found they are best used in person. On Echo the competition drive was missing. I don't feel I gained as much, so I chose to attend in person.
	Still very effective and helpful. Seeing material in question form always helps even though the competitive aspect is taken out of it. (*also fits under Review, Repeat, Practice, Helpful, Positive*)

## Discussion

As described by Karasik [47], teaching is like salesmanship; you need to catch the buyer’s attention before you can make a sale! Engaged teaching helps you capture students’ attention, and make them receptive to, and involved with, the concepts. Student engagement will not occur if either motivation or active learning, which interact synergistically, are missing [21]. Motivation is the feeling of interest or enthusiasm that makes somebody want to do something, and is the portal to engagement [21]. As such, methods that improve or maintain motivation in students are critical. Our results indicate that the microbiology TP ARS games were motivating because of the variety of interactions, and their engagement and learning features.

### Game design and variety

Varied learning opportunities and methods are critical to maintaining students’ attention [48]. It makes intuitive sense that variety will help prevent burnout with ARS. Three of the survey categories had items that addressed variety. *The variety of games we played helped me stay interested and focused* was the second highest rated learning item (Fig. 4), and *I enjoy the variety of interactions* was the third highest rated engagement item (Fig. 3). When students were asked to look at photographs of the different types of TP interactions that had been used over the year, and rate the extent to which each was engaging, *The variety provided by all of the different types of TP interactions* was rated very or somewhat engaging by 91 % of the respondents (Fig. 2). These results support our hypothesis that students would value the variety of TP interactions.

### Engagement

Competition is certainly motivating, and is a critical game dynamic [20, 37]. Friendly peer competition was tied for the most highly rated engagement aspect of the TP ARS games (Fig. 3). Other engagement features of the TP ARS games that were highly rated were the perceived rise in energy level, the variety of interactions, and being on their toes and in game mode with their clickers (Fig. 3). Approximately two-thirds of the respondents reported that during TP games they enjoyed holding the clickers and got an emotional lift [42]. While many educators attribute the success of ARS to the interaction between pedagogy and ARS use, rather than the clicker per se, our results reveal a role for the device itself as a contributing factor to the positive perceptions.

When students were asked which TP environment they learned best in, individual competition (one clicker per student), team competition (one clicker per student), team competition (one clicker per team), or no competition, we were not surprised to find a clear preference for competition, especially individual competition (53 % of the students selected individual competition, one clicker per student; 33 % selected team competition, one clicker per student). These are, after all, highly motivated medical students! Of more interest was what these responses may reveal about the preference for holding clickers themselves during team play. The high value the students gave the clickers in the set of questions querying engagement parameters (Fig. 3) support this finding.

Slightly more than half of the respondents were focused on the prize during TP games, our lowest positive engagement response (Fig. 3). The games were low-stakes, no points, just trivial prizes related to topics covered; for example, candy, travel size disinfectant bottles, travel size toothbrushes, and, occasionally, a raffle for a book or flashcard set. The survey results indicate that the students were much more motivated by the competition than the prizes. The games were anonymous, but there was social pressure to pay attention and respond correctly.

Chance is an essential part of a fun game because chance means uncertainty, and uncertainty means surprises [20]. According to Schell [20], risk and randomness are like spices; a sprinkling of them brings out the flavor of everything else in your game. In this study, surprise Wagering slides were occasionally placed prior to the last question of the session. Students had to quickly decide whether to wage all or part of their points on the next, unseen question. Seventy-four percent of the respondents found the Wagering slides very or somewhat engaging (Fig. 2). Other surprise elements included random placement of Fastest Responder slides, with the fastest correct responder winning an immediate prize. Fastest Responder game interactions were rated the lowest; even so, 70 % of the respondents found these interactions very or somewhat engaging (Fig. 2). Fastest Responder interactions were strict recall. As such, a possible explanation for this result is that students placed less value in these short-stem, non-clinical vignette questions. Alternatively, some students may need more time to think through even short-stem questions since the material has just been presented. Since approximately half of the respondents agreed or strongly agreed that they were focused on the prize, some students may have more negative impressions of the Fastest Responder interactions because they were never able to think quickly enough to win a Fastest Responder prize.

Flow is a construct used to operationalize engagement [43–45]. Flow is being fully emerged in learning and forgetting everything around oneself; absorption and enjoyment are at the core of the flow construct [43–45]. The engagement prompts comprising the flow element were, *I am focused on the activity, My energy level rises,* and *I get an emotional lift* (Fig. 3). The majority of the students responded positively to these prompts, revealing that flow contributes to positive perceptions of engagement with gamified TP interactions.

ARS fosters social cohesion in large group classrooms through shared knowledge of its members (viewing clicker data over time, how well others are doing, what peers think), by giving each student a voice, and by breaking the class into smaller, more cohesive groups [49]. The data generated by ARS communicate information to students about their classmates, whether the questions have a single correct answer, or are being used to spark discussion [49]. In addition to fostering social cohesion with team competitions and peer teaching slides, our TP interactions naturally incorporated the game mechanic social fabric. Social fabric is the idea that people like one another better after they’ve played games together, build a higher level of trust, and have a greater willingness to work together [20, 37]. While we did not query student perception of social fabric, we hope that the gamified TP interactions this class experienced during their first year of medical school had a positive influence on camaraderie.

### Learning

The feeling that one’s learning activities are purposeful and rewarding is another driving force for motivation [50]. In the current study, most students perceived that they gained practice applying theoretical knowledge to clinical scenarios, prioritized concepts they needed to review, made connections among complex concepts, gained an interest in microbiology, learned valuable concepts from more knowledgeable peers, and were challenged (Fig. 4). The feedback provided by the clicker questions gave students an opportunity to develop familiarity with summative instruments because the majority of the questions in the TP games matched the style used in our summative assessments, with clinical vignette stems and multiple choice answers. This question type mimics the United States medical licensing exams. Games have been shown to help counter feelings of despair related to assimilating large volumes of facts and terminology [24]. Medical microbiology is a difficult, content-dense discipline, so the fact that the majority of the respondents gained an interest in the subject is remarkable.

In a study involving more than 6,000 undergraduates in various disciplines, females reported greater learning and engagement with ARS [51]. In another study involving 410 undergraduates using ARS in physics courses, learning gains and positive perceptions of ARS were greater for females than males [52]. Given these results, we predicted that there would be significant differences between responses from our male and female students. However, only one question on the survey showed a difference in responses between males and females, *I prioritized the concepts I needed to review* (Fig. 4). Major differences between our study and the two discussed above is that our interactions were gamified, and they involved medical students, not undergraduates. Furthermore, the specific survey item in the present study was not queried in the undergraduate ARS studies.

We are not aware of any literature regarding student’s use of TP questions after class. More than half of the respondents provided written responses to the prompt, *After TP games, my strategy for follow-up studying involves…*The coded responses indicate that many students made the extra effort to view the TP interactions again, for practice, on Echo360 (Table 1). The TP questions were not provided before or after the in-class games. Responses that clearly indicated students were specifically using the TP questions fell into two coding categories, Focus on Weak Areas and Review the Game (Table 1). The positive response to the learning query *I gained an interest in microbiology* (Fig. 4), suggests that the gamified TP interactions may have helped motivate students to prepare for microbiology exam questions. Due to additional variables that could affect outcomes, exam performance for the Class of 2017 cannot be compared to previous classes that did not experience TP ARS interactions.

The overwhelmingly positive responses to the gamified TP interactions, including *I gained an interest in microbiology*, suggest that students had positive academic emotions related to medical microbiology. This is an intriguing possibility, given that academic emotions are related to processes associated with academic performance [53]. Future studies will need to investigate any potential academic gains with gamified TP interactions.

A small percent of the participants were not engaged and did not place learning value in the TP ARS interactions (Figs. 2, 3 and 4). This result is not surprising; it’s difficult to satisfy all learning styles with a single teaching method. In addition, it’s possible that these students think that clickers and games are gimmicky, although they did not indicate this in their open-ended responses. Alternatively, these students could have negative feelings about the instructor or the discipline.

### Value via lecture capture

According to open-ended responses, using lecture capture (Echo360) to review gamified TP presentations also had learning and engagement value (Table 2). Students valued the ability to go at their own pace, pause the presentation to think through questions, and review the questions again. Five students wrote comments indicating they were engaged, even in this mode. However, eight students commented that the TP presentations were not as engaging when viewed in Echo360. Whether students would have different perceptions of reviewing ‘standard’ TP presentations on Echo360, without the added variety and game elements, is unknown.

A handful of recent studies have compared perceptions of ARS when students are on a main campus or at a distant campus. In an undergraduate health information management course with nine on-campus and six distance education students using lecture capture software, two of the distance education students disliked the ARS because they were not able to interact and could only view the class discussion and not take part in it [54]. Overall, more on-campus students than distance education students had positive perceptions of the learning and interactive aspects of ARS. More recently, the vLink feature of TurningPoint was used to aggregate real-time data from multi-campus sites via clickers and USB receivers [55]. In this study, a pharmacy course using ARS was broadcast via synchronous, interactive video to two distant sites. The majority of the students (177 respondents) believed that ARS made it easier to participate (85.3 %) and helped them focus (75.7 %) when the lecturer was physically at a different campus, but student perceptions of ARS were significantly more positive from students at the site with the live lecturer.

### Limitations

This research is limited by the institutional and cultural contexts in which it was conducted. The outcomes of this study may have been influenced by student perceptions of the instructor, the nominal prizes awarded during some of the TP ARS interactions, the types of questions incorporated in the games, the anonymous play mode, the relative paucity of technical difficulties due to the author’s (RKP) previous experience with ARS, and the purchase of clickers by SOMA instead of by the students. Surveys were collected anonymously in order to reduce the likelihood of response bias. We were not able to characterize non-responders, who may or may not have favorable impressions of TP games. The results of this study might not be generalizable to other disciplines, younger learners, less motivated learners, or cultures that place more value on non-interactive lectures. In attempting to apply these methods and findings toward an innovation in a different context, investigators should consider the specific constraints, type of game employed, outcome measures used, and the natural environment of the study setting.

## Conclusions

Our hypothesis was that the medical students would be engaged by the variety of TP interactions offered during 22 medical microbiology presentations. The significant majority of students surveyed in this study were engaged by the variety of TP games, and gained an interest in microbiology, making the endeavor well worth the effort. The TP ARS games appear to be an extremely useful classroom tool for medical microbiology.

## Additional file

Additional file 1:
**Microbiology Turning Point Games Survey.**
