# Supplementary material for: Student perceptions of gamified audience response system interactions in large group lectures and via lecture capture technology
Source: BMC Med Educ. 2015 May 22;15:92. doi: 10.1186/s12909-015-0373-7 (PMC4477320; doi:10.1186/s12909-015-0373-7)
Supplement: Additional file 1: — Microbiology Turning Point Games Survey. [file 12909_2015_373_MOESM1_ESM.pdf]

Microbiology TurningPoint Games Survey 2014

Dear Students,

Thank you for providing feedback regarding Pettit microbiology Turning Point games.

All responses are anonymous.

Responses from this survey will be used for educational research and the improvement of the curriculum.

The responses will not affect your grade.

There are no foreseeable risks or discomforts to you in SOMA's sharing the aggregate results of this educational research.

Any publishable findings will be reported in aggregate format.

For questions about this research project, please contact Robin Pettit, Ph.D.

1. What is your age range?

- ◯ 22-25
- ◯ 26-30
- ◯ 31-35
- ◯ 36-40
- ◯ 41+

2. What is your gender?

- ◯ Male
- ◯ Female

3. I typically experienced TurningPoint presentations by this professor____________.

- ◯ In person
- ◯ Via lecture capture (Echo 360)

Other (please specify)

Microbiology TurningPoint Games Survey 2014

4. LEARNING. To what extent did TP games foster learning? During TurningPoint games...

	Strongly Agree	Agree	Neutral	Disagree	Strongly Disagree
1. I made connections among complex concepts.	○	○	○	○	○
2. I prioritized the concepts I need to review.	○	○	○	○	○
3. I practiced applying theoretical knowledge to clinical scenarios.	○	○	○	○	○
4. I was challenged and stretched beyond my comfort level.	○	○	○	○	○
5. I learned valuable concepts from more knowledgeable peers.	○	○	○	○	○
6. I gained an interest in microbiology.	○	○	○	○	○
7. The variety of games we played helped me stayed interested and focused.	○	○	○	○	○

5. Engagement/Flow: Please rate the extent to which you agree with the following statements. During TurningPoint games...

	Strongly Agree	Agree	Neutral	Disagree	Strongly Disagree
1. My energy level rises.	○	○	○	○	○
2. I get an emotional lift.	○	○	○	○	○
3. I enjoy the variety of interaction (individual, team, wagering, Mystery Bug, etc.)	○	○	○	○	○
4. I enjoy friendly peer competition.	○	○	○	○	○
5. I am focused on the activity.	○	○	○	○	○
6. I enjoy holding game devices such as clickers.	○	○	○	○	○
7. Using clickers puts me in game mode.	○	○	○	○	○
8. Using clickers in class keeps me on my toes.	○	○	○	○	○
9. I am focused on the prize.	○	○	○	○	○

6. Look at the photographs of the different types of TP interactions. Please rate the extent to which each activity is engaging.

	Very	Somewhat	Neutral	Not very	Not at all	I did not experience this activity.
Fastest Responder	◯	◯	◯	◯	◯	◯
Custom Correct Answer Indicator	◯	◯	◯	◯	◯	◯
Clearing up Misconceptions	◯	◯	◯	◯	◯	◯
Mystery Bug	◯	◯	◯	◯	◯	◯
Activating Previous Knowledge	◯	◯	◯	◯	◯	◯
Wagering	◯	◯	◯	◯	◯	◯
Ranking Responses	◯	◯	◯	◯	◯	◯
Most Valuable Player	◯	◯	◯	◯	◯	◯
Peer Teaching	◯	◯	◯	◯	◯	◯
The variety provided by all of the different types of TP interactions	◯	◯	◯	◯	◯	◯

Comments

7. In which of the following TP environments do you learn the best?

- ☐ Individual competition (one clicker per student)
- ☐ Team competition (one clicker per student)
- ☐ Team competition (one clicker per team)
- ☐ No competition

8. After TurningPoint games, my strategy for follow up studying involves:

9. Describe the learning value, if any, of viewing these TurningPoint presentations on Echo.
